# eDNA metabarcoding reveals differences in fish diversity and community structure in heterogeneous habitat areas shaped by cascade hydropower

**DOI:** 10.1002/ece3.10275

**Published:** 2023-07-07

**Authors:** Ruli Cheng, Yang Luo, Yufeng Zhang, Qinghua Li, Yingwen Li, Yanjun Shen

**Affiliations:** ^1^ Laboratory of Water Ecological Health and Environmental Safety, School of Life Sciences Chongqing Normal University Chongqing China

**Keywords:** cascade hydropower, environmental DNA, fish diversity, species composition of the community, the Wujiang River

## Abstract

Freshwater ecosystems are under great threat from humans, among which habitat heterogeneity is the most obvious, being one of the important reasons for the decline of fish diversity. This phenomenon is particularly prominent in the Wujiang River, where the continuous rapids of the mainstream have been divided into 12 mutually isolated sections by 11 cascade hydropower reservoirs. Based on the fact that conventional survey methods are more harmful to the ecological environment, the efficient and noninvasive environmental DNA metabarcoding (eDNA) approach was used in this study to conduct an aquatic ecological survey of the 12 river sections of the mainstream of the Wujiang River. A total of 2299 operational taxonomic units (OTUs) were obtained, corresponding to 97 species, including four nationally protected fish species and 12 alien species. The results indicate that the fish community structure of the Wujiang River mainstream, which was originally dominated by rheophilic fish species, has been changed. And there are differences in fish species diversity and species composition among the reservoir areas of the mainstream of the Wujiang River. The fish species in the area have gradually declined under the influence of anthropogenic factors such as terraced hydropower and overfishing. The fish populations consequently have demonstrated a tendency to be species miniaturized, and the indigenous fish are severely threatened. In addition, the fish composition monitored by the eDNA approach was found to be close to the fish composition of historical information on the Wujiang River, indicating that eDNA approach may be used as a complementary tool to conventional methods in this basin.

## Graphical abstract

Freshwater ecosystems are under great threat from humans, among which habitat heterogeneity is the most obvious, being one of the important reasons for the decline of fish diversity. This phenomenon is particularly prominent in the Wujiang River, where the continuous rapids of the mainstream have been divided into 12 mutually isolated segments by 11 cascade hydropower reservoirs. Based on the fact that traditional survey methods are more harmful to the ecological environment, the efficient and noninvasive environmental DNA metabarcoding (eDNA) technology was used in this study to conduct an aquatic ecological survey of the 12 river sections of the mainstream of the Wujiang River.

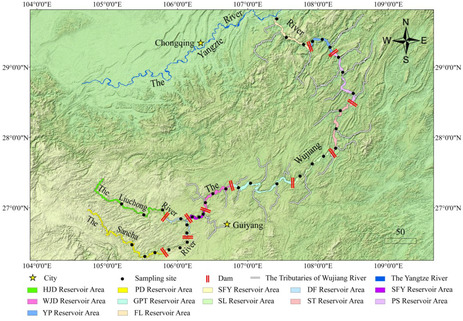

## 1 INTRODUCTION

Biodiversity is necessary to maintain the stability of ecosystem function, where changes in diversity caused by species distribution can also lead to changes in ecosystems (McKenzie et al., 1989; Oliver et al., 2015; Pecl et al., 2017). Fish community diversity is an important part of biodiversity, which not only regulates the stability and resilience of aquatic ecosystems but also serves as a key indicator for evaluating its health (Holmlund & Hammer, 1999; Zou et al., 2020). Continuous river habitats and the length of free‐flowing rivers are vital for aquatic organisms, especially fish (Liermann et al., 2012). The fragmentation of river habitats caused by the development of cascade hydropower can lead to a decline in fish biodiversity (Banks et al., 2013). The connectivity of the rivers is blocked by the dams of the cascade hydropower, which impedes fish migration in addition to impacting fish habitat due to changes in hydrological conditions, thereby also affecting fish reproduction (Cao, 2017; Cheng et al., 2015; Liermann et al., 2012). Changes in the aquatic environment are closely related to the ecological composition of fish populations and as advanced predators in the aquatic environment, the stability and sustainability of fish populations under cascade hydropower development are of great concern to scholars (Li et al., 2020; Nobile et al., 2019).

As the largest tributary on the right bank of the upper Yangtze River, the Wujiang River originates in the foothills of the Wumeng Mountains and flows through Yunnan, Guizhou, Chongqing, and Hubei, finally flowing into the Yangtze River in the Fuling District of Chongqing City (Liang et al., 2017; Qiu et al., 2022). Since the construction of the Wujiangdu Hydropower station in 1970, the mainstream of the Wujiang River has been developed to 11 cascade hydropower levels. With the adoption of the head‐to‐tail approach by the main‐stem power stations of the Wujiang River, the natural flowing water habitat was gradually transformed into a continuous or discontinuous slow‐flowing lake and reservoir habitat. Such modification has determined changes in the food chain, biodiversity, community structure, and resources of the aquatic ecosystem, with a particularly pronounced impact on the fish community (Xiong et al., 2021). These changes not only alter the composition and community structure of fish species, but fish migration pathways are also blocked by habitat fragmentation caused by intensive hydropower development in the basin, leading to the decline or even disappearance of some short‐distance migratory fish species (Yao et al., 2009). Additionally, the genetic exchange between fish populations in different habitats is also blocked (Li et al., 2020; Liermann et al., 2012). Comprehensive fishery data for the mainstream of the Wujiang River have been so far concentrated on individual reservoirs, thereby not fully reflecting the changes in the composition of fish species and species diversity in the context of the existing hydropower development (Wang et al., 2001; Xiong et al., 2021; Yang et al., 2010, 2019). There is, therefore, a need to understand the current situation of the overall fish diversity in the study area and take reasonable measures to reduce the adverse effects caused by water conservancy projects to further protect the fish diversity in the Wujiang River basin.

In the past, fish surveys in the Wujiang River were carried out by conventional methods such as netting, cage catching, and electric fishing (Xiao et al., 2015; Yang et al., 2010, 2019). These methods are not only time‐consuming and labor‐intensive, and sensitivity is also limited, such as low capture rates for benthic species by electric fishing, but also cause irreversible damage to fish and the ecosystem (Jerde et al., 2011; Shen et al., 2022). In addition, information on fish size, distribution, and density may be assessed using acoustic devices such as fish finders (Jiang et al., 2016; Sun et al., 2014). However, this method cannot identify species. This study, therefore, used environmental DNA metabarcoding (eDNA metabarcoding) of water samples to supplement the conventional survey data. The mixed DNA produced by organisms is preserved, extracted, amplified, sequenced, and classified using eDNA metabarcoding approach to determine the distribution of organisms in the environment (Deiner et al., 2015; Stewart, 2019; Taberlet, Coissac, Hajibabaei, & Rieseberg, 2012). This technique is a useful tool for studying the distribution of aquatic and terrestrial organisms and their biodiversity (Doi et al., 2017, 2021). eDNA approach is currently widely used in the study of fish communities (Bohmann et al., 2014; Doi et al., 2017; Fujii et al., 2019; Hänfling et al., 2016). Due to the varied characteristics and habitats of fish, none of the conventional capture methods can completely detect fish species (Fujii et al., 2019). Scholars, therefore, mostly choose the efficient and noninvasive eDNA approach in combination with conventional methods. Multiple studies have shown that the performance of eDNA in fish detection is similar to or higher than conventional methods (Hänfling et al., 2016; Shaw et al., 2016; Yamamoto et al., 2017).

To explore the changes in fish species composition and species diversity in the context of the existing hydropower development in the Wujiang River, in July 2021, environmental DNA metabarcoding of water samples was used to conduct comprehensive and systematic monitoring of fish species composition in 12 river sections of the mainstream of the Wujiang River. This is to enable further measures to protect fish diversity in the region, as well as to provide basic information for the evaluation of the impact of the construction of hydropower plants on the composition of fish diversity in future.

## 2 MATERIALS AND METHODS

### 2.1 Study area

**FIGURE 1:**
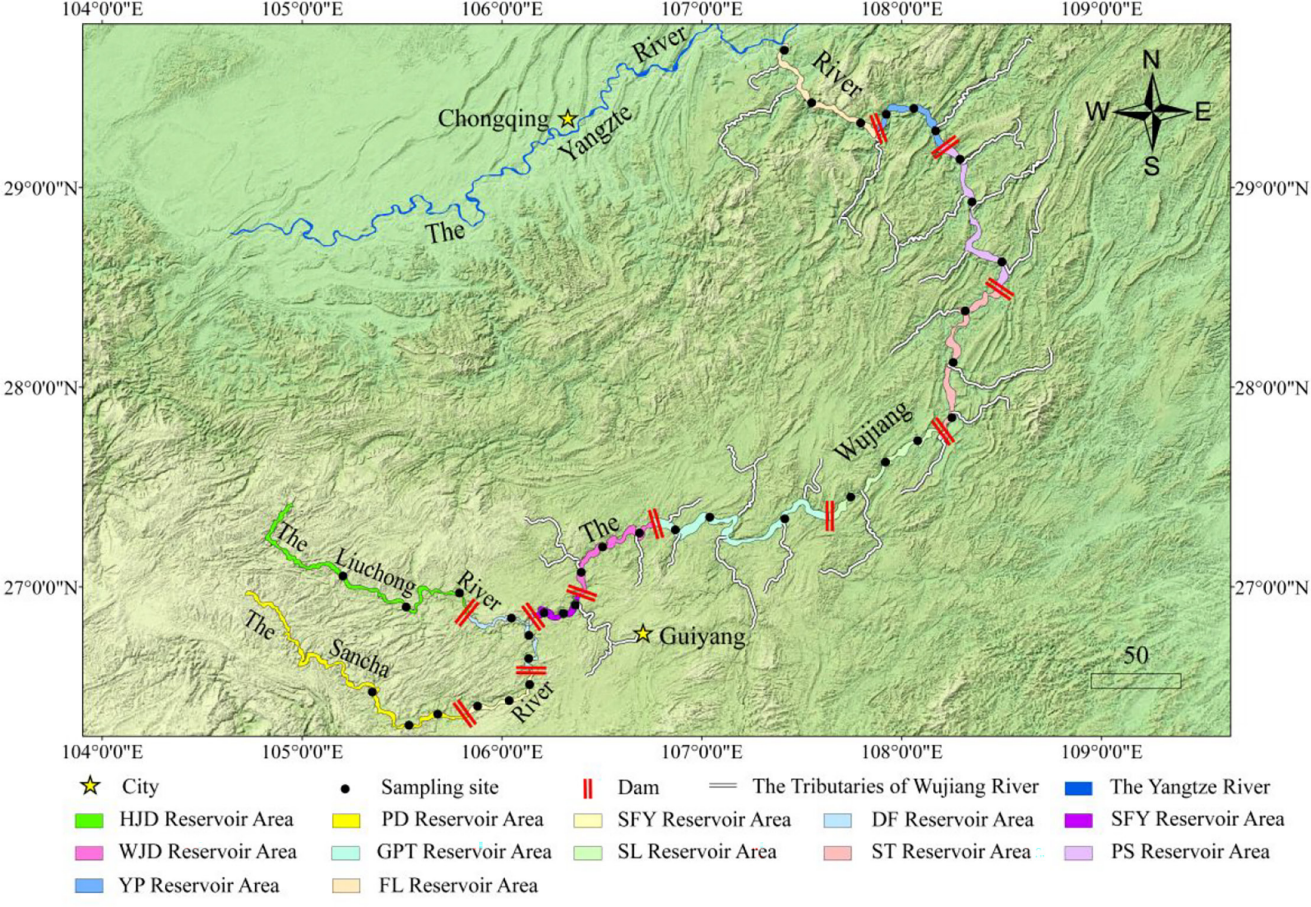
Information on sampling locations by ArcGIS 10.7 (Refer to Section 2.1 for the explanation about these acronyms in the figure).

As a mountain river with a basin area of 87,920 km^2^, a natural drop of 2124 m, and an annual runoff of 53.4 billion m^3^, the Wujiang River has several tributaries. At present, the mainstream of the Wujiang River is divided into 12 isolated sections by hydropower dams, the earliest of which was the Wujiangdu hydropower station built in 1970. In this study, the 12 river sections in the mainstream of the Wujiang River (11 cascade hydropower reservoir areas and the section into the river from Fuling) were considered as 12 different sampling sections (Hongjiadu: HJD, Puding: PD, Yingzidu: YZD, Dongfeng: DF, Suofengying: SFY, Wujiangdu: WJD, Goupitan: GPT, Silin: SL, Shatuo: ST, Pengshui: PS, Yinpan: YP, Fuling: FL), and for each section, three sampling sites were located at the upper, middle and lower side, respectively (Figure 1).

### 2.2 Historical data and reference database

The list of all the fishes in the mainstream of the Wujiang River since 1989 was summarized from “Fishes of Guizhou Province” (Lv, 1989), “The Fishes of Sichuan, China” (Ding, 1994), and historical literatures (Dai & Li, 2006; Ding, 1994; Wang et al., 2001; Xiao et al., 2015; Yang et al., 2010, 2019; Appendix 1). The 12S rRNA and mitochondrial genome sequences of all freshwater fish species in the NCBI nucleotide database (https://www.ncbi.nlm.nih.gov/) were collected and used as a reference database for eDNA annotation in this study.

### 2.3 eDNA sampling and processing

In July 2021, 3 L of water samples were collected at each sampling site with a water collector approximately 0.5 m below the water surface. Before the collection of the water samples at different sampling sections, all the utensils were disinfected with a 10% bleach solution, followed by washing with distilled water and replacing the disposable utensils (Pilliod et al., 2013). The water samples from the same sampling section were mixed, and 6 L of the mixed water samples were divided into three technical replicate samples (every 2 L of mixed water was a technical replicate sample), and the remaining water samples were discarded. The collected water samples were immediately stored under refrigeration and pumped within 24 h onto mixed cellulose filter membranes (Whatman) with a pore size of 0.45 μm using a vacuum pump. If the water samples contained more sediments, they were prefiltered with sterile medical gauze during collection (Xu & Chang, 2016). The filtering equipment was sterilized before each sample was filtered to avoid cross‐contamination between samples. Negative control was set up with 2 L of distilled water to assess the presence of exogenous DNA contamination. The membranes were then stored frozen at −80°C for the next step of the DNA extraction.

PowerWater DNA Isolation Kits (Qiagen) were used to extract the total DNA captured in the filtration membranes. To reduce the degradation of DNA, a sterile TE solution was used instead of PW6 (sterile eluent without EDTA) for the final elution step. Finally, 1% gel electrophoresis was used to detect the quality of the extracted eDNA (Hao et al., 2018). Each sample was extracted independently, and a blank filter membrane was set synchronously as a negative control. The DNA samples were then stored at −20°C until PCR amplification.

Teleo2 primers were used to amplify the mitochondrial 12S rRNA gene fragment (tele02‐F: 5′‐AAA CTC GTG CCA GCC ACC‐3′; tele02‐R: 3′‐GGG TAT CTA ATC CCA GTT TG‐5′) (Taberlet, Coissac, Pompanon, et al., 2012). PCR amplification was performed using TransStart Fastpfu DNA Polymerase and a 20 μL reaction system, which included 4 μL 5× FastPfu Buffer, 2 μL dNTPs (2.5 mM), 0.4 μL FastPfu Polymerase, 1–2 μL template DNA (10 ng) and 0.8 μL each of upstream and downstream primers (5 μM). The system was finally made up to 20 μL with ddH_2_O. The PCR amplification procedure used is as follows: initial denaturation at 95°C for 5 min, followed by 35 cycles of denaturation at 95°C for 30 s, annealing at 58°C for 30 s, elongation at 72°C for 45 s, and final elongation at 72°C for 10 min. 3× PCR replicates were carried out for each sample, then replicate PCR products were pooled together before further processing. The PCR products were finally visualized using 2% agarose gel electrophoresis. In this study, detectable PCR products were obtained for all 36 samples, with no products for any of the negative controls. The PCR products were recovered by gel cutting using the AxyPrepDNA Gel Recovery Kit (AXYGEN), eluted with Tris HCl. At last, the purified products of PCR were sequenced using Illumina NovaSeq 6000 sequencing platform for high‐throughput sequencing.

### 2.4 Bioinformatic analyses and taxonomic assignment

The valid sequences of all samples were first obtained according to the barcode, and high‐quality reads are retained according to the following criteria by Trimmomatic v.0.36 (Bolger et al., 2014): Filter bases with a read tail quality value below 20, set a window of 10 bp, truncate back‐end bases from the window if the average quality value within the window is below 20, and remove reads <100 bp in length, and the paired reads were merged into one sequence using FLASH (Magoč & Salzberg, 2011). Then, the chimeras were removed using a combination of denovo and reference sequences using Usearch software and the GOLD database, and primers were removed by Cutadapt (v4.0, https://cutadapt.readthedocs.io/). The denovo sequences are available on NCBI (https://dataview.ncbi.nlm.nih.gov) under the following accession numbers SRR19906589‐SRR19906624 (Appendix 2). The Usearch software (version 10 http://drive5.com/uparse/) was finally used to conduct OTU clustering analysis according to sequence similarity ≥97% (Edgar, 2013). The OTU representative sequences were compared, classified, and annotated in the MitoFish database (http://mitofish.aori.u‐tokyo.ac.jp/) by using the Blastn tool and the uclust algorithm with identity value ≥97% and E‐value ≤10^−5^ (Sales et al., 2020). Sequences assigned to the same taxon were merged and only clusters of over 10 sequences and with OTUs assigned to fish were retained for downstream analyses. Fish OTUs were then compared with historical data from the Wujiang River basin to screen out fish that are unlikely to belong to the region.

### 2.5 Species composition and diversity analysis

The data (number of sequences) were randomly extracted from the samples in a certain amount of sequencing, their correspondent OTUs were counted, and a curve (Appendix 3) was constructed using the extracted amount of sequencing data with the corresponding representative OTUs for assessing the sampling depth of the samples. Reads from each sample were randomly selected using QIIME v.1.9.0 to normalize all eDNA sample data by the smallest number actually sequenced in all samples (Caporaso et al., 2010), which means that the lowest value of sequence abundance in all samples is used as the depth of draw level, and the sequences of all samples are randomly drawn to a uniform amount of data, that is, the total sequence abundance is the same across all samples. After standardization, the relative sequence abundance (read ratio) of each species in each sample was kept constant. For this study, species composition analysis, alpha diversity, and beta diversity analysis were performed based on the results of the OTU cluster analysis. The taxonomic information of fishes was improved by referring to the Fishbase database (https://www.fishbase.de/) and “Species Diversity and Distribution of Inland Fishes in China” (Zhang & Zhao, 2016), in addition to counting some ecological habits of various fishes. Based on the species’ relative sequence abundance, alpha diversity is the diversity of species within a relatively small area, often expressed as species richness, reflecting the results within a sample (Doi et al., 2021); Beta diversity analysis is the variation in species characteristics across sites, reflecting the relationships between samples (Doi et al., 2021). To explore the similarities in fish composition among the reservoir areas, in this study, the principal coordinates analysis (PCoA) as well as a dendrogram were constructed using Bray–Curtis distance matrices based on the relative sequence abundance of fish species in each river section. Finally, statistical analysis and figures were performed using R version 4.0.3 (https://www.r‐project.org/) (R Core Team, 2020).

Based on the relative sequence abundance of species, alpha diversity analysis of fish communities in the mainstream of the Wujiang River was conducted by calculating the Chao1 index (Chao, 1984), Shannon index (Shannon, 1948), Simpson index (Simpson, 1949), and Pielou index (Pielou, 1966). The Pielou index values of the three parallel samples for each river section satisfied the homogeneity of variance and normal distribution and one‐way analysis of variance (ANOVA) was used for significance analysis. By contrast, the Shannon, Simpson, and Chao1 indices did not satisfy the conditions of the ANOVA, therefore it was tested using nonparametric tests (the Kruskal–Wallis test). The *p* < .05 threshold was used in all cases to determine whether there was a significant difference in each index between the river sections. And the McNaughton index was used to determine the species with extremely high relative sequence abundance (Ling et al., 2022; Woodland et al., 2019). The Chao1 index is a measure of species richness (Chao, 1984). The Shannon index measures community diversity by taking the richness and evenness of the community into consideration, with higher index values indicating higher community diversity (Shannon, 1948). The Simpson index commonly represents an area's biodiversity, where higher values indicate lower community diversity (Simpson, 1949). The Pielou index is a measure of relative species richness in a community (Pielou, 1966). The indexes are calculated as follows:

(1) $$\text{Chao1 index}:S_{\text{Chao1}}=S_{\mathrm{obs}}+n_{1}\left(n_{1}-1\right)/2\left(n_{2}+1\right)$$

(2) $$\text{Shannon}–\text{Wiener index}:H^{\prime }=\sum P_{i}\log P_{i},P_{i}=n_{i}/N$$

(3) $$\text{Simpson index}:D=\left[\sum n_{i}\left(n_{i}-1\right)\right]/N\left(N-1\right)$$

(4) $$\text{Pielou index}:J=H/H_{\max}$$

(5) $$\text{McNaughton index}:Y_{i}=n_{i}/N\times f_{i}$$

In the formula: *S*_obs_ is the actual number of species; *n*_1_ is the number of species containing only one sequence; *n*_2_ is the number of species containing only two sequences; *N* is the total number of fish sequences detected; *n*_*i*_ is the number of sequences of the i‐th fish species; *H* is the Shannon index; *H*_max_ is the maximum Shannon index that can be achieved with the same species richness (i.e., when the abundance of all species in the community is identical); *Y*_*i*_ is the dominance index of the i‐th fish species (species with *Y*_*i*_ > 0.02 are dominant) and *f*_*i*_ is the frequency of occurrence of the *i*‐th frequency of occurrence of species *i*.

## 3 RESULTS

### 3.1 Sequence information and taxonomic assignment

After filtering for quality control, 5,115,906 valid sequences were obtained from 36 water samples, ranging from 503,602 to 747,755 sequences for each sample. When the threshold of 97% was set 2299 OTUs were obtained by clustering. A total of 533 OTUs were shared by 12 sampling sections, accounting for 23.18% of all OTUs (Appendix 4). Finally, as per the similarity of more than 97%, 2299 OTUs were clustered from the reference database and were assigned to 97 fish species belonging to six orders, 20 families, and 69 genera (Table 1).

**TABLE 1:** Species lists and sequence number of fish detected by eDNA at 12 sampling areas in the mainstream of the Wujiang River.

No.	Order	Family	Genus	Species	Ecological type			The number of fish sequences at each sampling site												Dominant index	The NCBI accession numbers
					Habitat water velocity	Habitat pelagic	Egg types	HJD	PD	YZD	DF	WJD	SFY	GPT	SL	ST	PS	YP	FL		
1	Acipenseriformes	Acipenseridae	*Acipenser*	*A. schrenckii*▲	F	B	A	66	82	14,625	64	35	54	37	34	29	29	38	30	0.00886822	MH973734.1 MH973733.1
2				*Acipenser* sp. *x Acipenser* sp. ▲	F	B	A	28	55	5339	26	18	28	12	18	20	16	1496	25	0.00415234	NC_036420.1 NC_063963.1
3	Cypriniformes	Catostomidae	*Myxocyprinus*	*M. asiaticus*●★	F	D_1_	A	0	1	0	1	0	1	0	1	1	0	0	1	0.00000176	AY986503.1 NC_006401.1
4		Cyprinidae	*Danio*	*D. rerio*▲	S	P_1_	D_3_	0	3	1	1	1	1	1	1	2	2	2	270	0.00015320	LC468887.1 OM236540.1
5			*Ctenopharyngodon*	*C. idellus*●	E	D_1_	D_2_	0	0	0	0	0	0	0	0	0	1	0	0	0.00000005	NC_010288.1 MG827396.1
6			*Squaliobarbus*	*S. curriculus*●	E	P_1_	D_3_	40	5519	48	31	31	45	37	163	29	38	82	1740	0.00457573	AP011218.1 KC351187.1
7			*Zacco*	*Z. platypus*●	F	B	D_3_	10	52	17	8	9	3	11	19	33	74	32	31	0.00017534	LC557065.1 LC557064.1
8			*Mylopharyngodon*	*M. piceus*●	E	D_1_	D_2_	3	1	2	1	2	2	12	2	3	6	19	63	0.00006802	MT084757.1 MF687137.1
9			*Opsariichthys*	*O. bidens*●■	F	P_1_	D_2_	11,182	4309	51,499	25,971	6728	25,867	379	12,058	3962	2499	445	369	0.08518608	DQ367044.1 MN832737.1
10			*Hemiculterella*	*H. sauvagei*●◆	F	P_1_	D_2_	30	35	27	28	30	37	29	29	35	3241	6692	465	0.00626165	NC_026693.1
11			*Hemiculter*	*H. leucisculus*●	S	P_1_	A	14	18	119	177	17	47	7	71	16	15	6	17	0.00030728	NC_022929.1 LC340359.1
12				*H. tchangi*●■◆	S	D_1_	A	87,709	11,086	129,051	258,695	24,252	39,116	9371	93,107	20,847	12,224	8097	17,888	0.41719473	NC_036740.1
13			*Pseudohemiculter*	*P. dispar*●	S	P_1_	A	2	4	7	17	1	3	2	10	1	11	15	249	0.00018882	NC_020435.1
14			*Ancherythroculter*	*A. nigrocauda*●◆	F	P_1_	A	1	8	7	8	9	3	7	2	1	7	6	8	0.00003929	NC_021414.1
15			*Pseudolaubuca*	*P. sinensis*●	E	P_1_	D_2_	0	3	1	1	1	1	0	1	1	1	7	18	0.00001881	NC_026712.1 AY050554.1
16				*P. engraulis*●	E	P_1_	D_2_	0	1	1	0	0	0	0	0	0	0	0	0	0.00000020	NC_020462.1
17			*Chanodichthys*	*C. ilishaeformis*	S	P_1_	A	3842	566	62	45	28	42	3317	43	32	30	36	64	0.00475400	NC_029722.1
18				*C. mongolicus*●	S	P_1_	A	66	106	90	100	68	59	81	107	69	204	2250	12,888	0.00943411	MZ032228.1 KF826087.1
19				*C. dabryi*●	S	P_1_	A	0	1	0	1	3	0	1	1	1	1	10	26	0.00001979	NC_021418.1 AP012111.1
20			*Culter*	*C. alburnus*●	S	P_1_	A	187	470	21	30	885	604	2532	29	20	35	52	137	0.00293320	MZ901180.1 KX244762.1
21			*Megalobrama*	*M. skolokovii*●	S	D_1_	A	13	13	15	16	3111	18	12	12	18	15	20	62	0.00194980	JX242528.1 NC_024422.1
22				*M. amblycephala*●	S	D_1_	A	2	1	0	1	4	2	3	5	1	2	11	134	0.00008923	MH289764.1 MH289763.1
23				*M. terminalis*●	S	D_1_	A	4	2	3	3	9	6	20	15	8	10	38	113	0.00012417	NC_018816.1 JX242530.1
24			*Distoechodon*	*D. tumirostris*●	F	D_1_	D_2_	10	18	16	16	17	15	2734	22	30	15	13	14	0.00156961	NC_011208.1
25			*Pseudobrama*	*P. simoni*●	E	D_1_	D_2_	2	2	3	11	2	4	2	11	3	1	6	98	0.00007794	AP011364.1 NC_022852.1
26			*Acheilognathus*	*A. barbatus*	S	D_1_	O	0	0	0	1	10	10	25	19	11	7	15	37	0.00005937	NC_026872.1
27				*A. rhombeus*	S	D_1_	O	0	0	0	0	0	0	2	1	0	1	4	12	0.00000489	LC146100.1 LC742196.1
28				*A. omeiensis*◆	S	D_1_	O	1	0	0	0	0	0	0	0	0	0	0	0	0.00000005	MG783572.1
29			*Rhodeus*	*R. sinensis*●	E	B	O	5863	2038	73	58	1131	360	47	92	742	50	57	58	0.00619773	NC_022721.1
30				*R. ocellatus*●	E	D_1_	O	9538	118	95	566	10,598	1659	73	1352	130	96	862	78	0.01475692	NC_011211.1 AB016697.1
31			*Spinibarbus*	*S. denticulatus*	F	D_1_	D_2_	0	0	0	0	2	0	0	0	0	1	5	30	0.00000743	NC_021616.1
32				*S. sinensis*●	F	B	D_2_	55	50	41	2311	82	1417	43	4363	1287	84	39	765	0.00617896	NC_022465.1 KC579368.1
33			*Acrossocheilus*	*A. yunnanensis*●	S	D_1_	A	0	0	0	65	0	2	0	0	2	1	0	1	0.00001735	MT476484.1 NC_028527.1
34				*A. monticolus*●◆	F	B	A	7	6	3	5	4	6	4	7	2153	7	5	7	0.00129830	LC146021.1 NC_022145.1
35			*Pseudogyrinocheilus*	*P. procheilus*●	F	D_1_	D_3_	5	10	7	230	4	100	5	8	2028	8	5	4	0.00141559	NC_024588.1
36			*Discogobio*	*D. yunnanensis*●	S	B	D_3_	182	29	2593	29	21	2294	20	19	2621	22	22	706	0.00501847	NC_025319.1
37			*Hemibarbus*	*H. labeo*●	F	D_1_	A	418	17	9	8	9	7	118	11	809	13	13	1198	0.00154225	ON479138.1 KP064328.1
38			*Bangana*	*B. rendahli*●◆	F	B	D_2_	0	0	1	0	0	0	0	0	1	0	0	0	0.00000020	LC278287.1
39				*B. tungting*●	F	B	D_2_	2	2	4	0	0	15	0	1	2	0	0	1	0.00000924	NC_027069.1
40			*Pseudorasbora*	*P. parva*●▲	E	D_1_	A	356	132	48	303	46	1718	56	51	41	52	66	1400	0.00250337	MH918118.1 LC742162.1
41			*Sarcocheilichthys*	*S. sinensis*●	E	D_1_	A	1	0	0	0	0	1	1	1	1	0	11	39	0.00001881	MN711646.1 KC847084.1
42				*S. nigripinnis*●	E	B	D_2_	0	1	0	1	3	1	6	3	2	2	6	17	0.00002052	NC_020608.1 KJ997940.1
43			*Coreius*	*C. heterokon*●	F	D_1_	D_2_	1	2	2	3	3	1	1	0	1	4	2	446	0.00025049	NC_020042.1
44			*Squalidus*	*S. argentatus*●	S	B	D_2_	6	13	7	8	8	7	6	8	8	16	2664	632	0.00198381	LC146038.1 KF819452.1
45			*Rhinogobio*	*R. typus*●	F	B	D_2_	2	4	3	2	1	5	6	3	4	5	5	862	0.00052894	LC458038.1 KU323963.1
46				*R. cylindricus*●◆	F	B	D_2_	0	0	0	0	0	1	0	0	0	0	0	0	0.00000005	NC_024540.1
47				*R. ventralis*●★◆	F	B	D_2_	6	5	5	4	4	3	4	2	3	3	5	1131	0.00068903	NC_022720.1
48			*Abbottina*	*A. rivularis*●	E	B	A	1	0	0	2	11	5	20	17	7	10	21	53	0.00007183	LC552373.1 LC069430.1
49			*Microphysogobio*	*M. kiatingensis*●	F	B	D_2_	6	7	5	12	6	8	6	6	6	23	3737	190	0.00235266	NC_037402.1
50			*Saurogobio*	*S. dumerili*●	E	D_1_	A	1	5	2	1	2	2	2	2	2	2	7	18	0.00002697	NC_022187.1
51			*Schizothorax*	*S. eurystomus*	F	B	A	31	43	30	13	24	109	32	35	53	26	42	32	0.00027561	ON920824.1
52				*S. davidi*●★	F	B	A	313	646	2461	2450	2644	643	6465	3254	5075	2616	1973	3276	0.01865711	MK861919.1
53			*Carassius*	*C. auratus*●	E	B	A	1609	207	156	161	107	1084	233	1847	749	354	860	228	0.00445376	LC069406.1 JN105355.1
54			*Cyprinus*	*C. carpio*●■	E	D_1_	A	18,947	6459	3740	5660	6968	241,884	3285	3843	5113	1835	3666	3803	0.17897299	NC_011192.1
55			*Procypris*	*P. rabaudi*●★◆	F	B	A	2	0	0	2	1	2	529	0	0	1	2	0	0.00018438	NC_010156.1 MW344880.1
56			*Hypophthalmichthys*	*H. molitrix*●	E	P_1_	D_2_	882	18	963	291	12	23	9	12	15	19	26	1089	0.00196974	MF180234.1 KJ756343.1
57			*Aristichthys*	*A. nobilis*●	E	P_1_	D_2_	36	2937	77	840	25	407	25	169	27	704	114	303	0.00332141	OQ793735.1 KM186182.1
58		Cobitidae	*Paramisgurnus*	*P. dabryanus*●	S	B	D_3_	14	1456	12	8	12	15	10	24	13	377	25	339	0.00135167	LC492322.1 MF579257.1
59			*Misgurnus*	*M. anguillicaudatus*●	E	B	A	96	304	8	1001	4	11	6	7	5	6	5	459	0.00112121	MN709617.1 KY307848.1
60			*Sinibotia*	*S. superciliaris*●	F	B	D_2_	5	9	5	7	7	6	6	6	8	6	794	1448	0.00135284	NC_030322.1
61				*S. reevesae*●◆	F	B	D_2_	1	2	1	1	1	2	2	1	1	1	218	138	0.00021638	AP011437.1 NC_026128.1
62			*Parabotia*	*P. fasciata*●	F	B	D_2_	2	8	5	2	4	3	5	2	4	5	8	728	0.00045505	NC_026130.1
63			*Leptobotia*	*L. taeniaps*●	F	B	D_2_	0	0	0	0	0	0	0	0	0	0	0	1	0.00000005	NC_019587.1
64			*Triplophysa*	*T. rosa*	F	B	D_3_	17	88	25	256	18	19	18	17	16	18	35	112	0.00037471	NC_019587.1
65				*T. stenura*	F	B	D_3_	5	16	6	3	2	3	3	4	2	3	8	13	0.00003988	NC_032692.1
66		Nemacheilidae	*Homatula*	*H. berezowskii*	F	B	A	5	880	7	10	4	3	0	4	4	10	4	102	0.00060576	NC_040302.1
67		Balitoridae	*Sinogastromyzon*	*S. szechuanensis*●◆	F	B	A	0	0	0	0	0	0	0	0	0	0	3	0	0.00000015	NC_036741.1 MW006667.1
68				*S. sichangensis*●◆	F	B	D_2_	11	16	13	376	16	15	13	17	16	21	3027	1704	0.00307570	NC_024534.1
69	Siluriformes	Ictaluridae	*Ictalurus*	*I. punctatus*▲■	E	B	A	1444	131	108	1830	35,949	117	169	102	158	99	107	98	0.02363921	LC552425.1 MF621722.1
70		Clariidae	*Clarias*	*C. gariepinus*	E	B	A	1532	14	12	7	5	13	4	7	4	8	6	7	0.00094939	LC036834.1 NC_027661.1
71		Siluridae	*Silurus*	*S. asotus*●	E	B	A	602	116	308	174	2629	540	95	3444	3196	81	73	117	0.00667037	MK895951.1 JX087351.1
72				*S. meridionalis*●	E	B	A	114	39	1022	35	5338	1238	25	608	33	35	2013	803	0.00662815	LC037013.1 JX087350.1
73		Bagridae	*Leiocassis*	*L. crassilabris*●	F	B	A	9	20	13	4	8	9	9	10	1204	12	3664	33	0.00292910	KC768227.1 NC_021394.1
74			*Pseudobagrus*	*P. pratti*●	F	B	A	3	6	1	3	1	2	2	1	1	2	3	4	0.00001701	NC_041443.1
75				*P. brevicaudatus*●	F	B	A	2	3	2	3	3	4	3	3	7	2	22	716	0.00045153	NC_021393.1
76				*P. medianalis*●◆	F	B	A	1	2	0	386	1	3	2	2	0	1	2	0	0.00017592	NC_037048.1
77			*Pelteobagrus*	*P. fulvidraco*●	S	B	A	1279	2700	196	1061	137	831	3229	165	1138	614	230	881	0.00730721	MK104136.1 MK104135.1
78				*P. vachelli*●	E	B	A	2	6	2	1	4	3	2	619	2	4	2	642	0.00075588	HM746660.1
79				*P. eupogon*●	S	B	A	1	5	2	1	10	12	30	20	15	11	18	50	0.00010262	NC_018768.1
80			*Hemibagrus*	*H. macropterus*●	S	B	A	0	5	1	0	0	0	1	0	0	1	1	0	0.00000220	NC_019592.1
81			*Glyptothorax*	*G. sinense*●	F	B	A	1	2	0	1	1	2	2	0	2	2	526	3	0.00026486	LC458102.1 NC_024672.1
82	Cyprinodontiformes	Poeciliidae	*Gambusia*	*G. affinis*●▲	S	P_1_	O	16	73	24	18	32	20	18	2089	22	36	21	38	0.00141148	LC193305.1 OL825609.1
83		Cyprinodontidae	*Oryzias*	*O. sinensis*	S	P_1_	A	0	0	1	1	0	0	1	1	0	0	9	26	0.00001143	NC_013434.1
84	Perciformes	Channidae	*Channa*	*C. maculata*	E	B	P_2_	11	3068	14	246	11	7	10	15	680	16	48	114	0.00248636	KC823606.1 JX978724.1
85		Centrarchidae	*Micropterus*	*M. salmoides*▲	S	D_1_	A	5	1975	11	3	4	10	5	6	4	6	8	463	0.00146602	LC474183.1 MH301076.1
86			*Lepomis*	*L. cyanellus*▲	S	D_1_	A	4	2	458	4	1	3	3	0	1	2	2	1	0.00025856	KP013087.1 OP422384.1
87		Cichlidae	*Oreochromis*	*O. niloticus*▲	E	B	O	0	0	0	0	0	0	0	0	0	0	1	1	0.00000020	GU477624.1 NC_013663.1
88			*Coptodon*	*C. zillii*▲■	E	B	O	1362	122	107	85	14,869	113	3885	16,926	135	196	98	89	0.02227582	C385286.1 MW194077.1
89		Gobiidae	*Rhinogobius*	*R.cliffordpopei*●■	E	B	O	2466	3254	55,653	20,082	24,175	11,700	10,404	22,720	580	2139	10,604	2597	0.09756278	MK288030.1 KP694000.1
90		Serranidae	*Siniperca*	*S. obscura*	E	B	P_2_	20	28	13	19	133	14	20	22	1058	34	5415	1412	0.00480150	NC_021136.1
91				*S. scherzeri*	E	B	P_2_	3	4	1	1	1	1	1	1	2	3	10	25	0.00003108	JQ010987.1 NC_015815.1
92				*S. undulata*●	E	B	P_2_	4	7	6	2	5	4	4	6	7	10	2226	14	0.00134580	NC_024433.1 KF815977.1
93				*S. roulei*	E	B	P_2_	2	3	1	1	0	0	1	1	1	0	1	3	0.00000616	KP710957.1 NC_024432.1
94		Eleotridae	*Micropercops*	*M. swinhonis*●	E	B	A	17	44	614	11	12	904	11	344	14	12	25	51	0.00120741	NC_021763.1 LC763826.1
95		Mastacembelidae	*Macrognathus*	*M. aculeatus*●	E	B	A	0	0	0	0	0	1	4	4	1	3	4	11	0.00000958	KT443991.1 NC_027435.1
96	Salmoniformes	Salangidae	*Protosalanx*	*P. chinensis*▲	S	P_1_	D_3_	0	0	1	11	1	0	0	0	0	0	0	0	0.00000191	MW291629.1 MH330683.1
97			*Neosalanx*	*N. tangkahkeii*▲	S	P_1_	D_3_	70	102	578	10,146	634	267	46	41	55	41	57	41	0.00708262	LC385223.1 NC_026455.1
Total	6	20	69	97				77	80	77	83	80	83	82	81	83	84	87	87		

*Note*: ●: Fish species in common with eDNA and historical data; ★: National protected fishes; ▲: Alien species; ■: The species with extremely high relative sequence abundance throughout the mainstream of the Wujiang River; ◆: The endemic fish of the upper Yangtze River; Dominant Index >0.02 is considered as species with extremely high relative sequence abundance.

Abbreviations: A, adhesive eggs; B, benthopelagic fishes; D_1_, demersal fishes; D_2_, drifting eggs; D_3_, demersal eggs; E, eurytopicity; F=flowing waters; O=other types; P_1_, pelagic fishes; P_2_, pelagic eggs; S, semi‐lentic water.

### 3.2 Differences in fish community among different river sections

**FIGURE 2:**
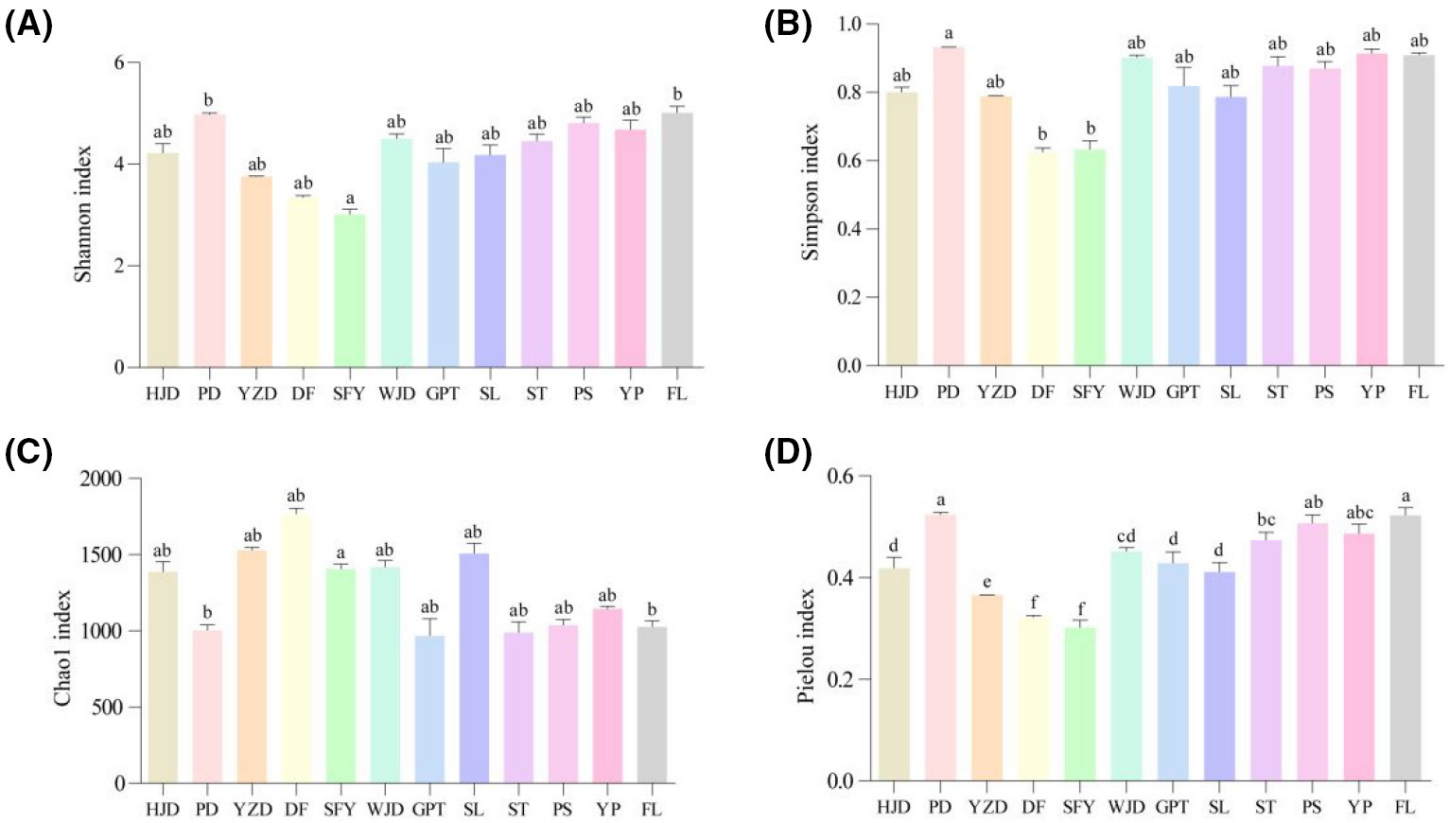
Significance histogram of each Alpha diversity index (The error bars show the absolute value of the standard error of the mean (SEM); on the error bars between different columns of the same small figure, the same small letter mean no significant difference (*p >* .05), while with different small letter mean significant difference (*p* < .05)). Graph A showing the Shannon index for each reservoir, graph B showing the Simpson index for each reservoir, graph C showing the Chao1 index for each reservoir and graph D showing the Pielou index for each reservoir.

As presented in the table (Appendix 5), the Simpson index and Pielou index were found to be the highest in the PD reservoir, the Chao1 index was the highest and the Simpson index was the lowest in the DF reservoir, the Shannon index was the highest in the FL river section, the Shannon index and Pielou index was the lowest in the SFY reservoir, and the Chao1 index was the lowest in the GPT reservoir. The Shannon index exhibited a statistically significant increase in both the FL river section and the PD reservoir compared to the SFY reservoir. Conversely, the Chao1 index displayed a statistically significant decrease in comparison to the SFY reservoir. Furthermore, the Simpson index demonstrated a statistically significant elevation solely in the PD reservoir when compared to both the DF and SFY reservoir. Notably, the Pielou index values were PD reservoir and FL river section HJD, GPT and SL reservoir YZD reservoir DF and SFY reservoir, and all were statistically significant (*p* < .05, Figure 2). In addition, the coverage of all river sections was between 0.999088 and 0.990211 (Appendix 5), indicating that the sequencing depth covered all the OTU data and could reflect the real diversity of the samples.

**FIGURE 3:**
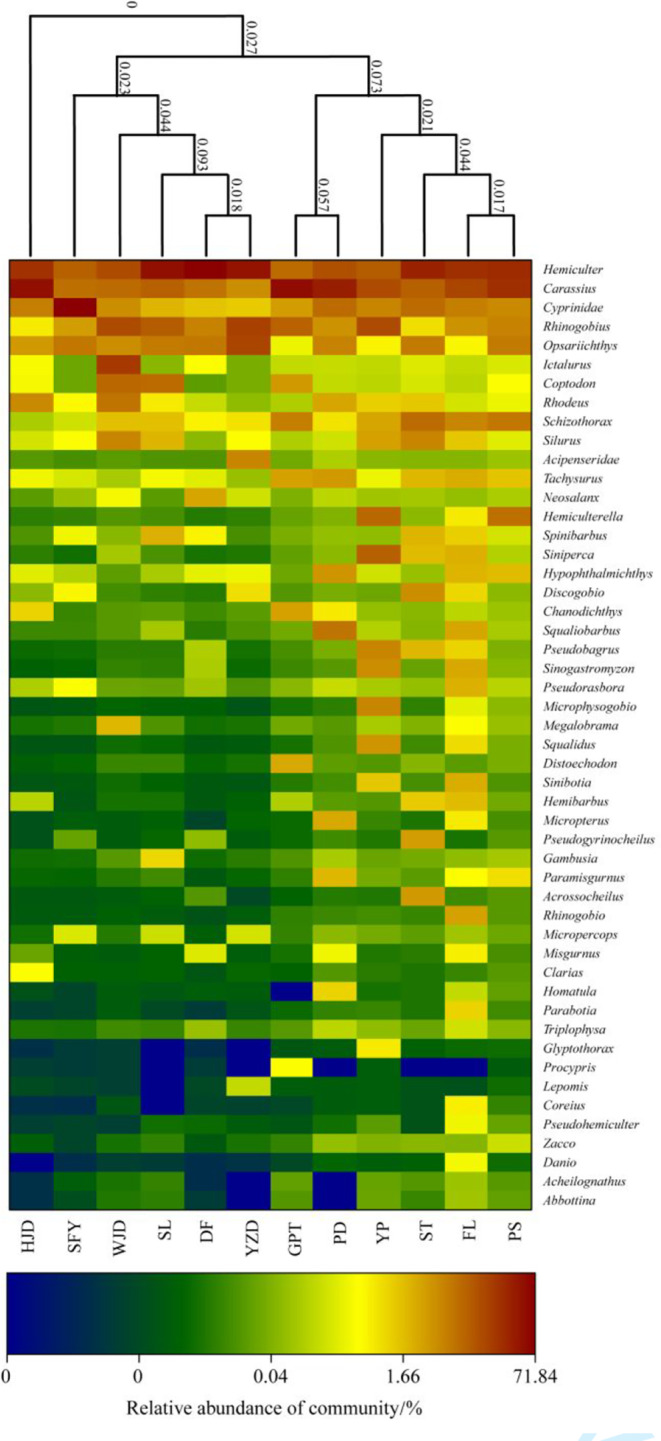
Species composition of fish at the genus level based on relative sequence abundance and the similarity tree of each river section (Refer to Section 2.1 for the explanation about these acronyms in the figure).

The total explanatory degree of the three PCoA axes was found to be 67.48% (Figure 3). From the two figures (Figure 3, Appendix 6), it may be observed that the most similar fish communities were those of DF and YZD, of GPT and PD, and of PS and FL. The fish composition of HJD, SFY, WJD, and SL reservoirs, on the contrary, was different from the other reservoir areas.

**FIGURE 4:**
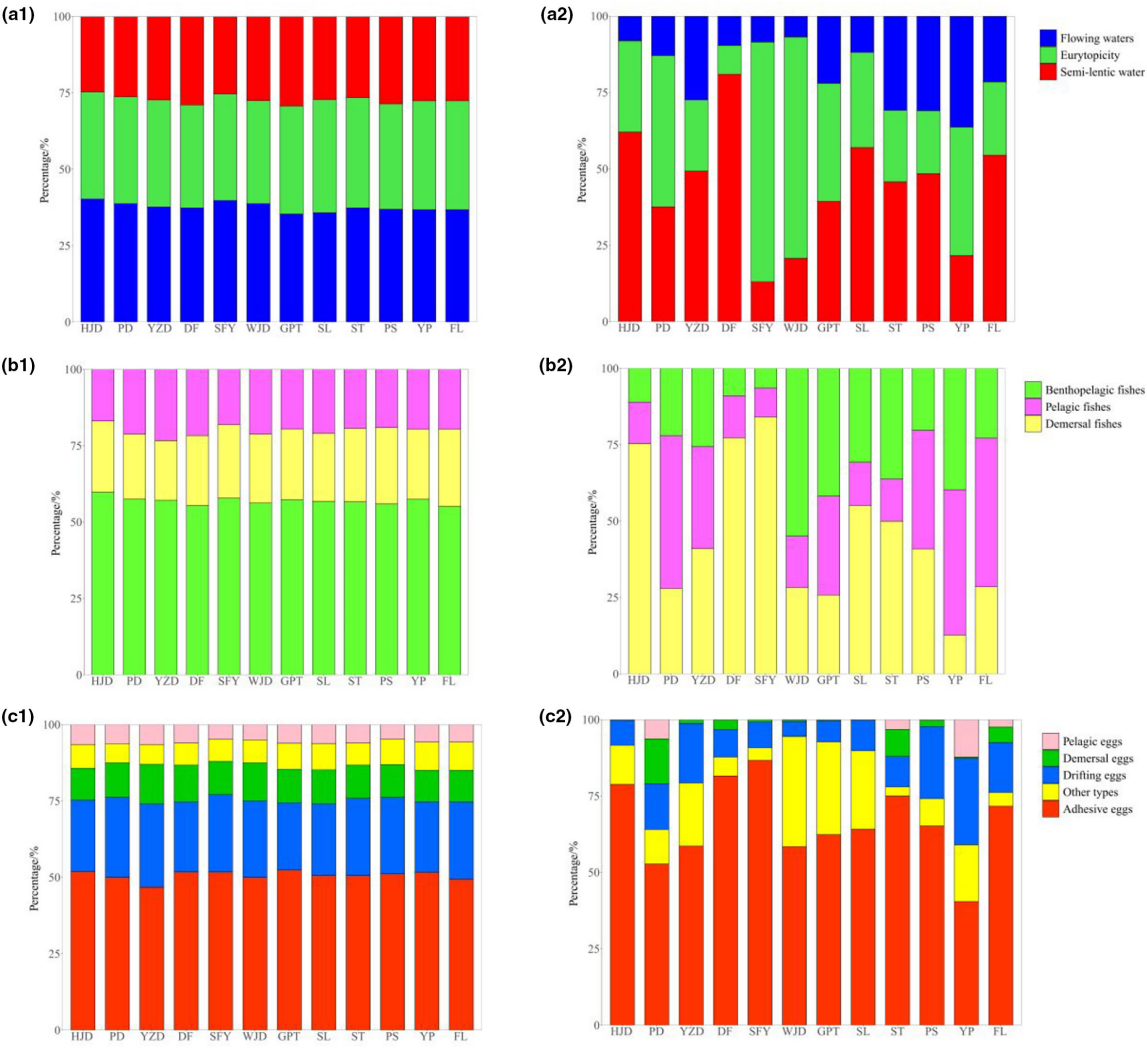
Composition of the ecological types in each reservoir area (a1: Habitat water velocity based on the number of species; a2: Habitat water velocity based on the number of relative sequences; b1: Habitat stratigraphy based on the number of species; b2: Habitat stratigraphy based on the number of relative sequences; c1: Spawning types based on the number of species; c2: Spawning types based on the number of relative sequences; refer to Section 2.1 for the explanation about these acronyms in the figure).

The results of this study explored the ecological types of fish in three aspects: water velocity, living water layer, and spawning type. Based on the presence of species, the composition of ecological types is relatively consistent across all river sections, with fishes that prefer flowing water (Figure 4a1), benthopelagic (Figure 4b1), and produced adhesive eggs (Figure 4c1) are the most predominant. However, the differences among river sections became prominent based on the relative sequence abundance. In terms of inhabiting water velocity, the relative sequence abundance of fishes preferring flowing water accounted for less, while the proportion of eurytopic or semi‐lentic water fishes increased, for example, HJD, PD, SFY (Figure 4a2). In terms of habitat water layer, six river sections have a high relative sequence abundance of demersal fish, three have a high relative sequence abundance of pelagic fish and three have a high relative sequence abundance of benthopelagic fish (Figure 4b2). In terms of spawning types, the relative sequence abundance was higher for fish producing adhesive eggs and lower for fish producing drifting eggs in all river sections (Figure 4c2).

### 3.3 The decline of indigenous fish species

In the eDNA results, 12 alien species such as *Micropterus salmoides*, *Coptodon zillii*, and *Lepomis cyanellus* were also detected, accounting for 12.37% of the total number of species in the survey (Table 1), of which all alien species except *Pseudorasbora parva* and *Gambusia affinis* were not recorded in the historical data. By contrast, historical data contain only six alien species, accounting for 3.11% (Appendix 1). What's more, seven river sections (PD, YZD, DF, WJD, GPT, SL and YP reservoir) with extremely high relative sequence abundance of fishes contained alien species.

By calculating the McNaughton index, we found that the fish species with extremely high relative sequence abundance in the Wujiang mainstream from the eDNA results were *H. tchangi*, *C. carpio*, *R. cliffordpopei*, *O. bidens*, *Ictalurus punctatus*, and *C. zillii* (Appendix 7), among these, the only rheophilic fish species is the *O. bidens*. The fish species with extremely high relative sequence abundance in each river section were shown in Appendix 7. Among the fishes with extremely high relative sequence abundance, the species counts of rheophilic fish in all river sections are <36%, except for the YZD reservoir, where the species counts of rheophilic fish account for 50% (Appendix 7). The sequence abundance of several previously common rheophilic fish such as *Coreius heterokon*, *Rhinogobio typus*, and *Rhinogobio cylindricus* was extremely low in this study (Table 1).

In addition, Xiao et al. (2015) surveyed 105 fish species in the Wujiang River downstream from 2006 to 2008 and 2011 to 2013, of which 103 species (98.10%) were indigenous and 25 species (23.81%) were endemic to the upper Yangtze River; Wang et al. (2023) surveyed 107 fish species in the Wujiang River basin during 2017–2021, including 94 indigenous fish species, accounting for 87.85%, and 15 endemic fish species in the upper Yangtze River, accounting for 14.02%. In this study, 97 fish species were detected in the mainstream of the Wujiang River, including 85 indigenous fish species, accounting for 87.63%, and 13 endemic fish species in the upper Yangtze River, accounting for 13.40%. The results imply that indigenous fish and endemic species of the Wujiang River are declining annually.

### 3.4 Comparing eDNA with historic records

**FIGURE 5:**
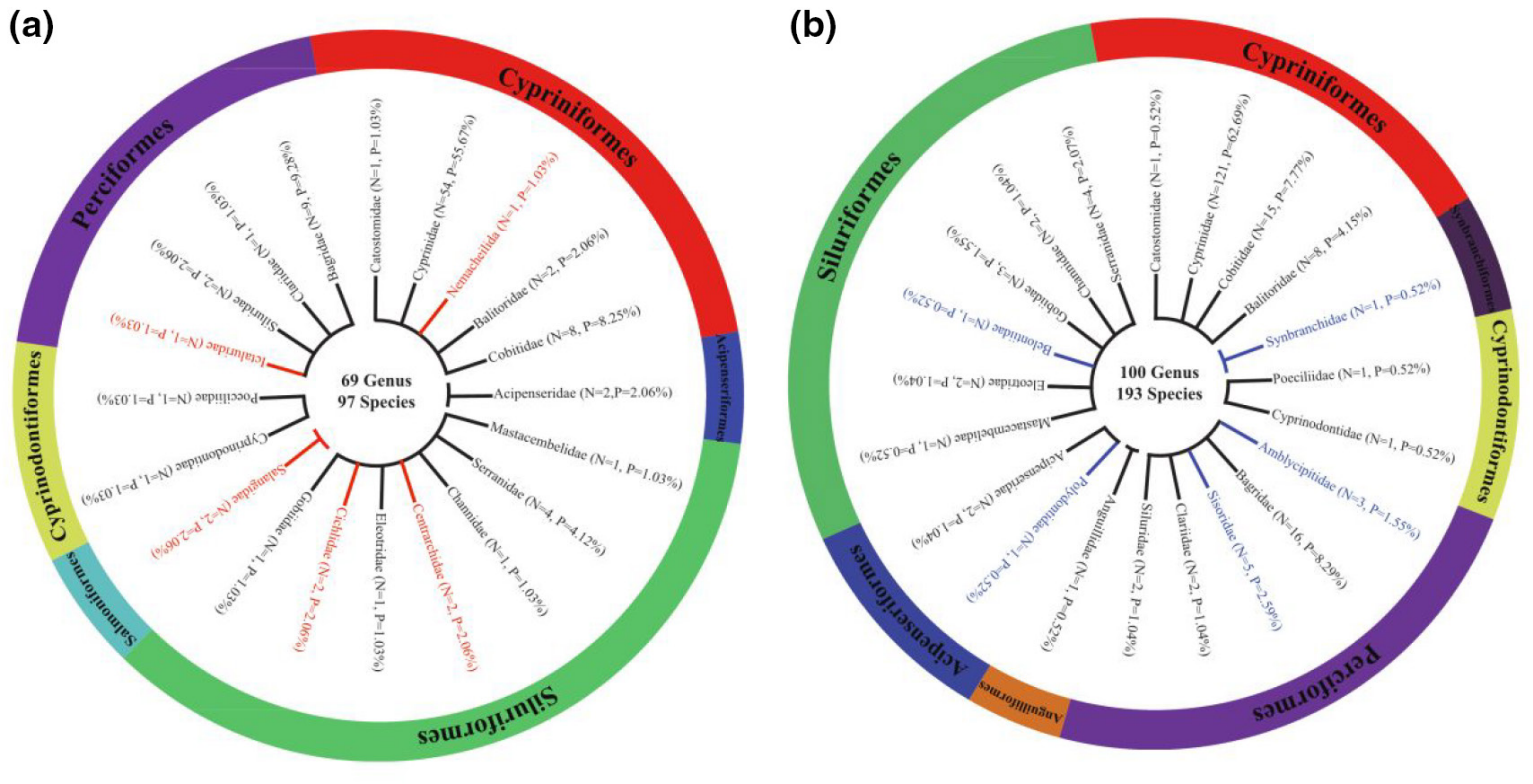
Family level composition map based on eDNA (a) and historical data (b) (Those in red are families that only exist in eDNA, and those in blue are families that only exist in historical data).

According to the historical data, 193 fish species belonging to seven orders, 20 families, and 100 genera occur in the mainstream of the Wujiang River, including 15 fish species of protection interest (Appendix 1). Among the historical data, Cyprinidae showed the highest proportion with 62.69%, *N* = 121; Bagridae the second with 8.29%, *N* = 16; Cobitidae the third with 7.77%, *N* = 15, while the other families accounted for a relatively small proportion (Figure 5b). Polydontidae, Anguillidae, Amblycipitidae, Sisoridae, Synbranchidae, and Belontiidae were only presented in historical data, not detected in eDNA results.

In the eDNA data, the relative sequence abundance and composition of each genus is presented in Figure 3, in which *Hemiculter*, *Carassius*, *Cyprinus*, *Opsariicjthys*, *Rhinogobius*, and other genera demonstrated extremely high relative sequence abundance in multiple rivers. On the contrary, *Abbottina*, *Acheilognathus*, *Danio*, and *Zacco* were found only in a few river sections with low relative sequence abundance. At the family level, the eDNA results were similar in composition to the historical data. Cyprinidae demonstrated absolute dominance, *N* = 54, accounting for 55.67%, followed by Bagridae, *N* = 9, accounting for 9.28%, and Cobitidae, *N* = 8, accounting for 8.25%, while the other families accounted for a small proportion (Figure 5a).

## 4 DISCUSSION

### 4.1 Differences in fish community among different river sections

As a whole, the alpha indices of each river section varied, with significant differences observed among some river sections, indicating differences in fish diversity among the river sections. These differences may stem as a result of the construction of individual dams, which block fish genetic exchange and cause changes in fish composition, leading to significant changes in fish diversity among river sections over time (Li et al., 2020; Yang et al., 2019). Among them, the FL river section demonstrated the highest Shannon index, indicating the highest diversity of the fish community in this river section, which may be related to the fact that this river section is the confluence of the Wujiang River and the Yangtze River. The fish in this river section can freely exchange genes with the fish in the mainstream of the Yangtze River, which is less affected by the cascade hydropower than other river sections. The Shannon index and Pielou index of the SFY reservoir were the lowest, indicating that the diversity of the fish community in this reservoir was the lowest, which may be related to the high abundance of some fish relative sequences in this reservoir. The Shannon index takes into account the richness and evenness of the community, therefore too high an abundance of individual fish relative sequences would cause the uneven distribution of species, in turn leading to lower diversity (Shannon, 1948; Wang et al., 2022). The Simpson index and Pielou index were found to be the highest in the PD reservoir, implying that the fish species’ relative sequence in the reservoir area was evenly distributed. However, the community richness was the lowest, which may be associated with the low concentration of eDNA. The PD reservoir is located in the upper reaches of the Wujiang River, where the sediment content in the water is high and the time of filtration is long, resulting in additional degradation of eDNA (Deiner et al., 2015). The DF reservoir demonstrated the lowest Simpson index, and the highest Chao1 index, indicating the highest fish community richness. This may also be associated with the fact that this reservoir is the confluence river section of the Sancha River and Liuchong River, and often the bait at the confluence of rivers is more abundant and more favorable for fish distribution. The GPT reservoir demonstrated the lowest Chao1 index, implying the lowest fish community richness, which may be associated with the fact that most of the sampling sites in this reservoir are piers highly influenced by human activities.

In this study, the beta diversity analysis demonstrated that the fish composition was similar in the DF and YZD reservoir, in the PS, ST, YP, and FL river sections, and in the GPT and PD reservoir. Research shows that the composition of the fish community changes with the increase in the reservoir operating time and is closely related to the habitat of tributaries, reservoir capacity, and flow velocity (Granzotti et al., 2018; Nilsson et al., 2005; Orsi & Britton, 2014). The similarity in the composition of the fish community between the DF and YZD reservoir may be because the two reservoir areas are adjacent to each other and were originally connected sections of the river before the formation of the dam. The PS, ST, YP, and FL sections were originally connected to rivers, and the three reservoir areas were formed more than a decade ago. The fish diversity and community structure in the reservoir may have gradually become stable (Xiong et al., 2021; Yang et al., 2019). The similarity of fish species composition in GPT and PD reservoirs may be due to similar tributary habitats in the two areas, which provide more alternative habitats for the original fish in the mainstream of the Wujiang River, thereby retaining a certain number of indigenous fish communities.

Based on the relative sequence abundance, the eDNA results demonstrated that the fish composition types in each reservoir area were still dominated by semi‐lentic water, and demersal species, which were quite different from the original fish composition structure in the Wujiang River. There are studies that show the mainstream of the Wujiang River was dominated by rheophilic fish, followed by eurytopic fish (Dan et al., 2004; Wang et al., 2023; Xiao et al., 2015). The decline of rheophilic fish species in the mainstream Wujiang River, which may be related to the development of cascade hydropower in Wujiang River. The construction of hydropower dams will usually lead to lower river flow velocity, sediment deposition, and more benthic bait organisms (Cao, 2019), which is more suitable for the survival of fish that prefer semi‐lentic water. On the contrary, the survival space of fish that prefer flowing water would be compressed, resulting in a decrease in numbers. The survival of fish with eurytopic and semi‐lentic water would show more advantages under these conditions. Xiao et al. (2015) found a decline in the fish species that have an affinity to flowing water in the lower reaches of the mainstream of the Wujiang River due to the construction of hydropower dams. Yang et al. (2019) found that the number of fish species above and below the dam decreased significantly after the impoundment of the YP reservoir, among which the number of fish preferring flowing water and laying drifting and adhesive eggs decreased further. Those are consistent with the results demonstrated in this survey.

### 4.2 The decline of indigenous fish species

Invasion of alien species greatly threatens the indigenous fish populations in the mainstream of the Wujiang River (Wang et al., 2023). The invasion is primarily caused by anthropogenic actions, such as escape from breeding farms, blind introduction, and blind release (Ba & Chen, 2012; Qiao et al., 2010; Wu et al., 2007). For example, *M. salmoides* and *Acipenser schrenckii* were previously cultured in cages in the mainstream of the Wujiang River, which may have resulted in the escape and subsequent invasion of the cultured species in the basin due to insufficient anti‐escape measures. In addition, the risk of the invasion of alien species is also increased by cascading hydropower. Reservoir storage and changes in hydrological conditions of river, the invading alien fish in the reservoir is superior in terms of competition for food bait and survival space when compared to indigenous fish, thus, leading to the reduction or even extinction of indigenous fish populations (Ba & Chen, 2012; Xiong, 2006). In this study, the results also indicate that the number of alien fish in the Wujiang River mainstream is gradually increasing, while the number of indigenous fish is decreasing annually. It is possible that this is the result of the alien fish encroaching on the survival space of indigenous fish.

In the result of eDNA, fish with extremely high relative sequence abundance and inhabiting stream velocity ecotypes based on relative sequence abundance in each river section show a lower proportion of rheophilic fish species, indicating that the fish community structure of the Wujiang River, which was originally dominated by rheophilic fish species, has changed (Dan et al., 2004; Wang et al., 2023; Xiao et al., 2015). As a typical mountain river, the Wujiang River has a large amount of flowing water habitat in its natural flow state, but the construction of the reservoir area has resulted in a drastic reduction of the original flowing water habitat, which has led to the reduction and even extinction of indigenous rheophilic fish populations (Wang et al., 2001; Xiao et al., 2015). Furthermore, we have found that the species with extremely high relative sequence abundance in the mainstream of the Wujiang River are now mostly small fishes such as *H. tchangi*, *R. cliffordpopei*, and *O. bidens*, which is also consistent with the findings in other river sections of the Yangtze River basin (Tan et al., 2017; Wei et al., 2021). This implies that the fish in the mainstream of the Wujiang River tend to be species miniaturization, which may be related to overfishing in the past. Some studies also show that several sections of the Wujiang River basin are in an overfished state (Xiao et al., 2015; Yang et al., 2019).

### 4.3 Comparing eDNA with historic records

In this study, 97 species of freshwater fishes were surveyed in the mainstream of the Wujiang River using eDNA approach. The detection rate was 50.26% compared with historical data, with the number of common species between the two being up to 72. Meanwhile, the results of the current survey were relatively similar to the historical data in terms of family level, all of which were overwhelmingly dominated by Cyprinidae, followed by Bagridae, and then Cobitidae. However, the six families Polydontidae, Anguillidae, Amblycipitidae, Sisoridae, Synbranchidae, and Belontiidae were only found in historical data, and five families of Nemacheilidae, Ictaluridae, Centrarchidae, Cichlidae, and Salangidae were only found in the present assay. It is possible that the families only present in historical data are too rare or even extinct and that the families that only appeared in the eDNA are alien species. The above results demonstrate that the eDNA survey results are close to the fish composition of historical information on the Wujiang River, reflecting the adaptability of eDNA approach in the Wujiang River basin. This may be used as an auxiliary tool to conventional methods (Jiang et al., 2016; Wang et al., 2022).

### 4.4 Impacts and conservation measures in the Wujiang River

The mainstream of the Wujiang River was divided into 12 isolated sections by 11 hydropower cascades. A series of unfavorable factors brought by cascade hydropower, including significant changes in hydrological conditions, changes in the natural runoff processes of the rivers, and stratification of water temperature, has led to a reduction in fish diversity in the basin, fish diversity and species composition in the 12 river sections produced differences, the tendency of fish to be miniaturized, and serious threat to the indigenous fish, which is consistent with several studies in the basin (Wei et al., 2019; Xiao et al., 2015; Yang et al., 2010, 2019). The following conservation recommendations are proposed based on the current situation of hydropower development and the diverse characteristics of fish communities in the mainstream of the Wujiang River:

1. Strengthening the protection of habitat. Establishing protected areas in situ is an effective way to conserve biodiversity (Andam et al., 2013). The National Natural Reserve Areas of Rare and Special Fishes of the Upper Yangtze River, established in the Yangtze River Basin in China, possesses a good fishery resource environment, and endemic fish recovery is also moving in a positive direction (Sun & Wang, 2020).
2. Regulating fishing. Under the context of Yangtze River preservation, a 10‐year “fishing ban” has been launched in the Yangtze River basin, which is of great significance in saving endangered aquatic life, maintaining biodiversity, and restoring the aquatic ecosystem (Cao, 2022).
3. Protecting tributary habitats. The habitat of the mainstream of the Wujiang River has been destroyed. Several fish migrate from the mainstream to the tributaries, and the fish species composition of some of the tributaries is very similar to that of the mainstream, which can be used as alternative habitats for conservation. For example, the Chishui River is one of the few large first‐order tributaries in the upper Yangtze River that still maintains its natural flow, providing the last refuge for rare and endemic fish species in the upper Yangtze River (Liu et al., 2020).
4. Enhancing artificial breeding. It is a good ecological compensation measure to aid in fish breeding using artificial technology and to help the fish population recover using proliferation and release. For example, the proliferation and release in the Silin reservoir of Wujiang had a value‐added effect on the population of *Barbodes sinensis* and *Varicorhinus simus* (Guo et al., 2018).
5. Establishing a database of fish diversity of the Wujiang River. The Wujiang River Basin lacks complete fish resource data post the construction of cascade hydropower. It is, therefore, necessary to establish a relevant database and set up multiple detection sites for long‐term, continuous and comprehensive monitoring of fish populations to facilitate subsequently targeted conservation of fish diversity in the Wujiang River Basin.

## 5 CONCLUSION

The results of this study demonstrate that with the impact of cascade hydropower, the fish composition and diversity of the mainstream of the Wujiang River have become significantly different among the river sections, while the fish populations of the basin are under the threat of severe species miniaturization and the sharp decline of indigenous fish.

## AUTHOR CONTRIBUTIONS

**Ruli Cheng:** Conceptualization (equal); data curation (equal); formal analysis (equal); investigation (equal); methodology (equal); resources (equal); visualization (equal); writing – original draft (equal); writing – review and editing (equal). **Yang Luo:** Data curation (equal); investigation (equal). **Yufeng Zhang:** Investigation (equal). **Qinghua Li:** Investigation (equal). **Yingwen Li:** Supervision (equal). **Yanjun Shen:** Investigation (equal); methodology (equal); supervision (equal); writing – review and editing (equal).

## FUNDING INFORMATION

This study was supported by the National Natural Science Foundation of China (Grant No. 32202939), and the Science and Technology Research Program of Chongqing Municipal Education Commission (Grant No. KJQN202100503).

## CONFLICT OF INTEREST STATEMENT

The authors declare that they have no conflict of interest.

## Data Availability

The reference sequences are available on NCBI (https://dataview.ncbi.nlm.nih.gov) under the following accession numbers SRR19906589‐SRR19906624.

## APPENDIX 1

### Species of catches monitored in the mainstream of the Wujiang River based on historical data

**Table:**

No.	Order	Family	Genus	Species
1	Acipenseriformes	Acipenseridae	*Acipenser*	*A. dabryanus*★
2				*A. sinensis*★
3		Polydontidae	*Psephurus*	*P. gladius*★
4	Anguilliformes	Anguillidae	*Anguilla*	*A. japonica*
5		Catostomidae	*Myxocyprinus*	*M. asiaticus*●★
6		Cyprinidae	*Luciobrama*	*L. macrocephalus*★
7			*Ctenopharyngodon*	*C. idellus*●
8			*Squaliobarbus*	*S. curriculus*●
9			*Elopichthys*	*E. bambusa*
10			*Ochetobibus*	*O. elongatus*
11			*Mylopharyngodon*	*M. piceus*●
12			*Phynchocypris*	*P. lagowskii*
13				*P. oxycephalus*
14			*Zacco*	*Z. platypus*●
15			*Aphyocypris*	*A. chinensis*
16			*Opsariicjthys*	*O. bidens*●
17	Cypriniformes		*Hemiculterella*	*H. sauvagei*●
18			*Hemiculter*	*H. leucisculus*●
19				*H. tchangi*●
20				*H. bleekeri*
21			*Pseudohemiculter*	*P. dispar*
22				*P. hainanensis*
23				*P. kweichowensis*
24			*Sinibrama*	*S. wui*
25				*S. macrops*
26				*S. taeniatus*
27			*Ancherythroculter*	*A. nigrocauda*●
28				*A. kurematsui*
29			*Pseudolaubuca*	*P. sinensis*●
30				*P. engraulis*●
31			*Chanodichthys*	*C. erythropterus*
32			*Culter*	*C. alburnus*●
33				*C. mongolicus*●
34				*C. dabryi*●
35				*C. oxycephalus*
36				*C. oxycephaloides*
37			*Megalobrama*	*M. pellegrini*●
38				*M. skolkovii*●
39				*M. terminalis*●▲
40			*Parabramis*	*P. pekinensis*
41			*Distoechodon*	*D. tumirostris*●
42			*Xenocypris*	*X. microlepis*
43				*X. argentea*
44				*X. davidi*
45			*Pseudobrama*	*P. simoni*●
46			*Paracheilognathus*	*P. meridianus*
47			*Acheilognathus*	*A. macropterus*
48				*A. tonkinensis*
49			*Rhodeus*	*R. lighti*
50				*R. sinensis*●
51				*R. ocellatus*●
52			*Barbodes*	*B. schwanenfdi*
53				*B. laticeps*
54			*Spinibarbus*	*S. hollandi*
55				*S. sinensis*●
56			*Percocypris*	*P. pingi*★
57			*Acrossocheilus*	*A. labiatus*
58				*A. monticolus*●
59				*A. parallens*
60				*A. yunnanensis*●
61			*Onychostoma*	*O. simum*
62				*O. angustistomata*★
63				*O. brevis*
64				*O. macrolepis*★
65				*O. barbata*
66				*O. lini*
67				*O. rara*
68				*O. ovalis rhomboides*
69			*Tor*	*T. brevifilis*
70			*Sinensia*	*S. multipunctatus*
71			*Sinilabeo*	*S. longibarbatus*
72				*S. tungting*●
73				*S. rendahli*●
74			*Sinocrossocheilus*	*S. guizhouensis*
75			*Rectoris*	*R. luxiensis*
76			*Pseudogyrincheilus*	*P. procheilus*●
77			*Garra*	*G. pingi*
78			*Discogobio*	*D. yunnanensis*●
79			*Hemibarbus*	*H. labeo*●
80				*H. maculatus*
81			*Belligobio*	*B. nummifer*
82			*Pseudorasbora*	*P. parva*●▲
83			*Sarcocheilichthys*	*S. sinensis*●
84				*S. parvus*
85				*S. kiansiensis*
86				*S. nigripinnis*●
87			*Gnathopogon*	*G. imberbis*
88				*G. herzensteini*
89				*G. sihuensis*
90			*Coreius*	*C. heterokon*●
91				*C. guichenoti*★
92			*Paracanthobrama*	*P. guichenoti*
93			*Squalidus*	*S. wolterstorffi*
94				*S. argentatus*●
95			*Platysmacheilus*	*P. exiguus*
96				*P. nudiventris*
97			*Rhinogobio*	*R. typus*●
98				*R. cylindricus*●
99				*R. hunanensis*
100				*R. ventralis*●★
101			*Abbottina*	*A. rivularis*●
102				*A. obtusirostris*
103			*Microphysogobio*	*M. fukiensis*
104				*M. tungtingensis*
105				*M. kiatingensis*●
106			*Saurogobio*	*S. dumerili*●
107				*S. dabryi*
108				*S. xiangjiangensis*
109				*S. gymnocheilus*
110			*Schizothorax*	*S. sinensis*
111				*S. kozlovi*
112				*S. wangchiachii*
113				*S. grahami*
114				*S. prenanti*
115				*S. davidi*●★
116				*S. griseus*
117			*Gobiobotia*	*G. filifer*
118				*G. meridionalis*
119			*Xenophysogobio*	*X. boulengeri*
120				*X. nudicorpa*
121			*Carassius*	*C. auratus*●
122			*Cyprinus*	*C. multitaeniata*▲
123				*C. carpio*●
124			*Procypris*	*P. rabaudi*●★
125			*Hypophthalmichthys*	*H. molitrix*●
126			*Aristichthys*	*A. nobilis*●
127		Cobitidae	*Cobitis*	*C. sinensis*
128			*Paramisgurnus*	*P. dabryanus*●
129			*Misgurnus*	*M. anguillicaudatus*●
130			*Botia*	*B. superciliaris*●
131				*B. pulchra*
132				*B. reevesae*●
133			*Parabotia*	*P. fasciata*●
134			*Leptobotia*	*L. elongata*★
135				*L. rubrilaris*★
136				*L. taeniaps*●
137				*L. microphthalrna*
138			*Paracobitis*	*P. wujiangensis*
139				*P. variegatus*
140				*P. potanini*
141			*Triplophysa*	*T. bleekeri*
142		Balitoridae	*Hemimzon*	*H. sinensis*
143			*Sinogastromyzon*	*S. szechuanensis*●
144				*S. sichangensis*●
145			*Lepturichthys*	*L. fimbriata*
146			*Vanmnenia*	*V. pinchowensis*
147			*Metahomaloptera*	*M. omeiensis*
148			*Beaufortia*	*B. szechuanensis*
149			*Jinshaia*	*J. sinensis*
150	Siluriformes	Clariidae	*Clarias*	*C. fuscus*▲
151				*C. batrachus*
152		Siluridae	*Silurus*	*S. asotus*●
153				*S. meridionalis*●
154		Bagridae	*Leiocassis*	*L. longirostris*
155				*L. crassirostris*
156				*L. crassilabris*●
157			*Pseudobagrus*	*P. albomarginatus*
158				*P. ussurirnsis*
159				*P. truncates*
160				*P. pratti*●
161				*P. brevicaudatus*●
162				*P. emarginatus*
163				*P. tenuis*
164				*P. medianalis*●
165			*Pelteobagrus*	*P. fulvidraco*●
166				*P. vachelli*●
167				*P. eupogon*●
168				*P. nitidus*
169			*Mystus*	*M. macropterus*●
170		Amblycipitidae	*Liobagrus*	*L. marginatus*
171				*L. nigricauda*
172				*L. marginatoides*
173		Sisoridae	*Pareuchiloglanis*	*P. feae*
174			*Euchiloglanis*	*E. davidi*★
175				*E. kishinouyei*
176			*Glyptothorax*	*G. fukiensis*
177				*G. sinense*●
178	Cyprinodontiformes	Poeciliidae	*Gambusia*	*G. affinis*●▲
179		Cyprinodontidae	*Oryzias*	*O. latipes*
180	Synbranchiformes	Synbranchidae	*Monopterus*	*M. albus*
181	Perciformes	Channidae	*Channa*	*C. asiatica*
182				*C. argus*
183		Serranidae	*Siniperca*	*S. scherzeri*
184				*S. undulata*●
185				*S. chuatsi*
186				*S. kneri*
187		Eleotridae	*Micropercops*	*M. cinctus*●
188			*Hypseleotris*	*H. swinhonis*
189		Godiidae	*Ctenogobius*	*C. cliffordpopei*●
190				*C. giurinus*
191			*Rhinogobius*	*R. giurinus*
192		Mastacembelidae	*Mastacembelus*	*M. aculeatus*●
193		Belontiidae	*Macropodus*	*M. opercularis*▲
Total	7	21	100	193

*Note:* ●: Fish species in common with eDNA and historical data; ★: National protected fishes; ▲: Alien species.

## APPENDIX 2

### The database address of the raw data and accession numbers

**Table:**

Accession numbers		BioProject		BioSample		Library ID	Files	
SRR19906624	https://dataview.ncbi.nlm.nih.gov/object/SRR19906624	PRJNA854355	https://dataview.ncbi.nlm.nih.gov/object/PRJNA854355	SAMN29425703	https://dataview.ncbi.nlm.nih.gov/object/SAMN29425703	HJD1	raw.split.HJD1.1.fq	raw.split.HJD1.2.fq
SRR19906623	https://dataview.ncbi.nlm.nih.gov/object/SRR19906623	PRJNA854355	https://dataview.ncbi.nlm.nih.gov/object/PRJNA854355	SAMN29425703	https://dataview.ncbi.nlm.nih.gov/object/SAMN29425703	HJD2	raw.split.HJD2.1.fq	raw.split.HJD2.2.fq
SRR19906612	https://dataview.ncbi.nlm.nih.gov/object/SRR19906612	PRJNA854355	https://dataview.ncbi.nlm.nih.gov/object/PRJNA854355	SAMN29425703	https://dataview.ncbi.nlm.nih.gov/object/SAMN29425703	HJD3	raw.split.HJD3.1.fq	raw.split.HJD3.2.fq
SRR19906601	https://dataview.ncbi.nlm.nih.gov/object/SRR19906601	PRJNA854355	https://dataview.ncbi.nlm.nih.gov/object/PRJNA854355	SAMN29425704	https://dataview.ncbi.nlm.nih.gov/object/SAMN29425704	PD1	raw.split.PD1.1.fq	raw.split.PD1.2.fq
SRR19906594	https://dataview.ncbi.nlm.nih.gov/object/SRR19906594	PRJNA854355	https://dataview.ncbi.nlm.nih.gov/object/PRJNA854355	SAMN29425704	https://dataview.ncbi.nlm.nih.gov/object/SAMN29425704	PD2	raw.split.PD2.1.fq	raw.split.PD2.2.fq
SRR19906593	https://dataview.ncbi.nlm.nih.gov/object/SRR19906593	PRJNA854355	https://dataview.ncbi.nlm.nih.gov/object/PRJNA854355	SAMN29425704	https://dataview.ncbi.nlm.nih.gov/object/SAMN29425704	PD3	raw.split.PD3.1.fq	raw.split.PD3.2.fq
SRR19906592	https://dataview.ncbi.nlm.nih.gov/object/SRR19906592	PRJNA854355	https://dataview.ncbi.nlm.nih.gov/object/PRJNA854355	SAMN29425705	https://dataview.ncbi.nlm.nih.gov/object/SAMN29425705	YZD1	raw.split.YZD1.1.fq	raw.split.YZD1.2.fq
SRR19906591	https://dataview.ncbi.nlm.nih.gov/object/SRR19906591	PRJNA854355	https://dataview.ncbi.nlm.nih.gov/object/PRJNA854355	SAMN29425705	https://dataview.ncbi.nlm.nih.gov/object/SAMN29425705	YZD2	raw.split.YZD2.1.fq	raw.split.YZD2.2.fq
SRR19906590	https://dataview.ncbi.nlm.nih.gov/object/SRR19906590	PRJNA854355	https://dataview.ncbi.nlm.nih.gov/object/PRJNA854355	SAMN29425705	https://dataview.ncbi.nlm.nih.gov/object/SAMN29425705	YZD3	raw.split.YZD3.1.fq	raw.split.YZD3.2.fq
SRR19906589	https://dataview.ncbi.nlm.nih.gov/object/SRR19906589	PRJNA854355	https://dataview.ncbi.nlm.nih.gov/object/PRJNA854355	SAMN29425706	https://dataview.ncbi.nlm.nih.gov/object/SAMN29425706	DF1	raw.split.DF1.1.fq	raw.split.DF1.2.fq
SRR19906622	https://dataview.ncbi.nlm.nih.gov/object/SRR19906622	PRJNA854355	https://dataview.ncbi.nlm.nih.gov/object/PRJNA854355	SAMN29425706	https://dataview.ncbi.nlm.nih.gov/object/SAMN29425706	DF2	raw.split.DF2.1.fq	raw.split.DF2.2.fq
SRR19906621	https://dataview.ncbi.nlm.nih.gov/object/SRR19906621	PRJNA854355	https://dataview.ncbi.nlm.nih.gov/object/PRJNA854355	SAMN29425706	https://dataview.ncbi.nlm.nih.gov/object/SAMN29425706	DF3	raw.split.DF3.1.fq	raw.split.DF3.2.fq
SRR19906620	https://dataview.ncbi.nlm.nih.gov/object/SRR19906620	PRJNA854355	https://dataview.ncbi.nlm.nih.gov/object/PRJNA854355	SAMN29425707	https://dataview.ncbi.nlm.nih.gov/object/SAMN29425707	SFY1	raw.split.SFY1.1.fq	raw.split.SFY1.2.fq
SRR19906619	https://dataview.ncbi.nlm.nih.gov/object/SRR19906619	PRJNA854355	https://dataview.ncbi.nlm.nih.gov/object/PRJNA854355	SAMN29425707	https://dataview.ncbi.nlm.nih.gov/object/SAMN29425707	SFY2	raw.split.SFY2.1.fq	raw.split.SFY2.2.fq
SRR19906618	https://dataview.ncbi.nlm.nih.gov/object/SRR19906618	PRJNA854355	https://dataview.ncbi.nlm.nih.gov/object/PRJNA854355	SAMN29425707	https://dataview.ncbi.nlm.nih.gov/object/SAMN29425707	SFY3	raw.split.SFY3.1.fq	raw.split.SFY3.2.fq
SRR19906617	https://dataview.ncbi.nlm.nih.gov/object/SRR19906617	PRJNA854355	https://dataview.ncbi.nlm.nih.gov/object/PRJNA854355	SAMN29425708	https://dataview.ncbi.nlm.nih.gov/object/SAMN29425708	WJD1	raw.split.WJD1.1.fq	raw.split.WJD1.2.fq
SRR19906616	https://dataview.ncbi.nlm.nih.gov/object/SRR19906616	PRJNA854355	https://dataview.ncbi.nlm.nih.gov/object/PRJNA854355	SAMN29425708	https://dataview.ncbi.nlm.nih.gov/object/SAMN29425708	WJD2	raw.split.WJD2.1.fq	raw.split.WJD2.2.fq
SRR19906615	https://dataview.ncbi.nlm.nih.gov/object/SRR19906615	PRJNA854355	https://dataview.ncbi.nlm.nih.gov/object/PRJNA854355	SAMN29425708	https://dataview.ncbi.nlm.nih.gov/object/SAMN29425708	WJD3	raw.split.WJD3.1.fq	raw.split.WJD3.2.fq
SRR19906614	https://dataview.ncbi.nlm.nih.gov/object/SRR19906614	PRJNA854355	https://dataview.ncbi.nlm.nih.gov/object/PRJNA854355	SAMN29425709	https://dataview.ncbi.nlm.nih.gov/object/SAMN29425709	GPT1	raw.split.GPT1.1.fq	raw.split.GPT1.2.fq
SRR19906613	https://dataview.ncbi.nlm.nih.gov/object/SRR19906613	PRJNA854355	https://dataview.ncbi.nlm.nih.gov/object/PRJNA854355	SAMN29425709	https://dataview.ncbi.nlm.nih.gov/object/SAMN29425709	GPT2	raw.split.GPT2.1.fq	raw.split.GPT2.2.fq
SRR19906611	https://dataview.ncbi.nlm.nih.gov/object/SRR19906611	PRJNA854355	https://dataview.ncbi.nlm.nih.gov/object/PRJNA854355	SAMN29425709	https://dataview.ncbi.nlm.nih.gov/object/SAMN29425709	GPT3	raw.split.GPT3.1.fq	raw.split.GPT3.2.fq
SRR19906610	https://dataview.ncbi.nlm.nih.gov/object/SRR19906610	PRJNA854355	https://dataview.ncbi.nlm.nih.gov/object/PRJNA854355	SAMN29425710	https://dataview.ncbi.nlm.nih.gov/object/SAMN29425710	SL1	raw.split.SL1.1.fq	raw.split.SL1.2.fq
SRR19906609	https://dataview.ncbi.nlm.nih.gov/object/SRR19906609	PRJNA854355	https://dataview.ncbi.nlm.nih.gov/object/PRJNA854355	SAMN29425710	https://dataview.ncbi.nlm.nih.gov/object/SAMN29425710	SL2	raw.split.SL2.1.fq	raw.split.SL2.2.fq
SRR19906608	https://dataview.ncbi.nlm.nih.gov/object/SRR19906608	PRJNA854355	https://dataview.ncbi.nlm.nih.gov/object/PRJNA854355	SAMN29425710	https://dataview.ncbi.nlm.nih.gov/object/SAMN29425710	SL3	raw.split.SL3.1.fq	raw.split.SL3.2.fq
SRR19906607	https://dataview.ncbi.nlm.nih.gov/object/SRR19906607	PRJNA854355	https://dataview.ncbi.nlm.nih.gov/object/PRJNA854355	SAMN29425711	https://dataview.ncbi.nlm.nih.gov/object/SAMN29425711	ST1	raw.split.ST1.1.fq	raw.split.ST1.2.fq
SRR19906606	https://dataview.ncbi.nlm.nih.gov/object/SRR19906606	PRJNA854355	https://dataview.ncbi.nlm.nih.gov/object/PRJNA854355	SAMN29425711	https://dataview.ncbi.nlm.nih.gov/object/SAMN29425711	ST2	raw.split.ST2.1.fq	raw.split.ST2.2.fq
SRR19906605	https://dataview.ncbi.nlm.nih.gov/object/SRR19906605	PRJNA854355	https://dataview.ncbi.nlm.nih.gov/object/PRJNA854355	SAMN29425711	https://dataview.ncbi.nlm.nih.gov/object/SAMN29425711	ST3	raw.split.ST3.1.fq	raw.split.ST3.2.fq
SRR19906604	https://dataview.ncbi.nlm.nih.gov/object/SRR19906604	PRJNA854355	https://dataview.ncbi.nlm.nih.gov/object/PRJNA854355	SAMN29425712	https://dataview.ncbi.nlm.nih.gov/object/SAMN29425712	PS1	raw.split.PS1.1.fq	raw.split.PS1.2.fq
SRR19906603	https://dataview.ncbi.nlm.nih.gov/object/SRR19906603	PRJNA854355	https://dataview.ncbi.nlm.nih.gov/object/PRJNA854355	SAMN29425712	https://dataview.ncbi.nlm.nih.gov/object/SAMN29425712	PS2	raw.split.PS2.1.fq	raw.split.PS2.2.fq
SRR19906602	https://dataview.ncbi.nlm.nih.gov/object/SRR19906602	PRJNA854355	https://dataview.ncbi.nlm.nih.gov/object/PRJNA854355	SAMN29425712	https://dataview.ncbi.nlm.nih.gov/object/SAMN29425712	PS3	raw.split.PS3.1.fq	raw.split.PS3.2.fq
SRR19906600	https://dataview.ncbi.nlm.nih.gov/object/SRR19906600	PRJNA854355	https://dataview.ncbi.nlm.nih.gov/object/PRJNA854355	SAMN29425713	https://dataview.ncbi.nlm.nih.gov/object/SAMN29425713	YP1	raw.split.YP1.1.fq	raw.split.YP1.2.fq
SRR19906599	https://dataview.ncbi.nlm.nih.gov/object/SRR19906599	PRJNA854355	https://dataview.ncbi.nlm.nih.gov/object/PRJNA854355	SAMN29425713	https://dataview.ncbi.nlm.nih.gov/object/SAMN29425713	YP2	raw.split.YP2.1.fq	raw.split.YP2.2.fq
SRR19906598	https://dataview.ncbi.nlm.nih.gov/object/SRR19906598	PRJNA854355	https://dataview.ncbi.nlm.nih.gov/object/PRJNA854355	SAMN29425713	https://dataview.ncbi.nlm.nih.gov/object/SAMN29425713	YP3	raw.split.YP3.1.fq	raw.split.YP3.2.fq
SRR19906597	https://dataview.ncbi.nlm.nih.gov/object/SRR19906597	PRJNA854355	https://dataview.ncbi.nlm.nih.gov/object/PRJNA854355	SAMN29425714	https://dataview.ncbi.nlm.nih.gov/object/SAMN29425714	FL1	raw.split.FL1.1.fq	raw.split.FL1.2.fq
SRR19906596	https://dataview.ncbi.nlm.nih.gov/object/SRR19906596	PRJNA854355	https://dataview.ncbi.nlm.nih.gov/object/PRJNA854355	SAMN29425714	https://dataview.ncbi.nlm.nih.gov/object/SAMN29425714	FL2	raw.split.FL2.1.fq	raw.split.FL2.2.fq
SRR19906595	https://dataview.ncbi.nlm.nih.gov/object/SRR19906595	PRJNA854355	https://dataview.ncbi.nlm.nih.gov/object/PRJNA854355	SAMN29425714	https://dataview.ncbi.nlm.nih.gov/object/SAMN29425714	FL3	raw.split.FL3.1.fq	raw.split.FL3.2.fq

## APPENDIX 5

### Alpha diversity index of each sampling area

**Table:**

Sample	Shannon	Simpson	Pielou	Chao1	Coverage
HJD	4.221249	0.800507	0.418321	1386.789	0.997708
PD	4.978286	0.932146	0.523679	1002.934	0.995078
YZD	3.755793	0.789178	0.365283	1529.871	0.998856
DF	3.340451	0.624704	0.321548	1765.366	0.998928
SFY	3.014909	0.633253	0.301602	1405.063	0.999088
WJD	4.503837	0.901877	0.451029	1418.693	0.997738
GPT	4.037468	0.818946	0.428127	967.7418	0.994465
SL	4.18036	0.787004	0.410552	1507.314	0.998053
ST	4.456131	0.877752	0.473167	989.7934	0.995410
PS	4.814697	0.870271	0.506504	1037.969	0.990211
YP	4.682676	0.914492	0.486484	1143.408	0.995264
FL	5.011128	0.908928	0.522378	1025.61	0.996095

## APPENDIX 7

### Dominant species in each river section and the whole mainstream of the Wujiang River

**Table:**

Sampling sites	Dominant species (McNaughton dominance index)	Total	Proportion of rheophilic fish species
HJD	*H. tchangi* (0.582278), *C. carpio* (0.125780), *O. bidens* (0.074251)□, *R. ocellatus* (0.063315), *R. sinensis* (0.038915), *C. ilishaeformis* (0.025499)	6	20.00%
PD	*H. tchangi* (0.224299), *C. carpio* (0.130346), *S. curriculus* (0.108911), *O. bidens* (0.086971)□, *R. cliffordpopei* (0.066047), *C. maculata* (0.061904), *A. nobilis* (0.059295), *P. fulvidraco* (0.054439), *R. sinensis* (0.041128), *M. salmoides* (0.039844), *P. dabryanus* (0.029378)	11	9.09%
YZD	*H. tchangi* (0.477085), *R. cliffordpopei* (0.205697), *O. bidens* (0.190350)□, *A. schrenckii* (0.054055)□	4	50.00%
DF	*H. tchangi* (0.774249), *O. bidens* (0.077725)□, *R. cliffordpopei* (0.060168), *N. tangkahkeii* (0.030360)	4	25.00%
WJD	*I. punctatus* (0.254801), *H. tchangi* (0.172055), *R. cliffordpopei* (0.171418), *C.zillii* (0.105394), *R. ocellatus* (0.075117), *C. carpio* (0.049419), *O. bidens* (0.047732)□, *S. meridionalis* (0.037835), *M. skolokovii* (0.022053)	9	11.11%
SFY	*C. carpio* (0.724852), *H. tchangi* (0.117284), *O. bidens* (0.077520)□, *R. cliffordpopei* (0.035120)	4	25.00%
GPT	*R. cliffordpopei* (0.217919), *H. tchangi* (0.196434), *S. davidi* (0.135247)□, *C.zillii* (0.081313), *C. ilishaeformis* (0.069395), *C. carpio* (0.068781), *P. fulvidraco* (0.067589), *D. tumirostris* (0.057198)□, *C. alburnus* (0.052951)	9	22.22%
SL	*H. tchangi* (0.553639), *R. cliffordpopei* (0.135236), *C.zillii* (0.100617), *O. bidens* (0.071737)□, *S. sinensis* (0.025943)□, *C. carpio* (0.022867), *S. asotus* (0.020471)	7	28.57%
ST	*H. tchangi* (1.148898), *C. carpio* (0.281616), *S. davidi* (0.279139)□, *O. bidens* (0.218274)□, *S. asotus* (0.175936), *D. yunnanensis* (0.144220), *A. monticolus* (0.118483)□, *P. prochilus* (0.111604)□, *S. sinensis* (0.070826), *L. crassilabris* (0.066295)□, *P. fulvidraco* (0.062553), *S. obscura* (0.058205), *H. labeo* (0.044539)□, *C. auratus* (0.041274), *R. sinensis* (0.040815), *C. maculata* (0.037385), *R. cliffordpopei* (0.032799)	17	35.29%
PS	*H. tchangi* (0.432538), *H. sauvagei* (0.114419), *S. davidi* (0.092361)□, *O. bidens* (0.088315)□, *R. cliffordpopei* (0.076080), *C. carpio* (0.064927), *A. nobilis* (0.024823), *P. fulvidraco* (0.021705)	8	25.00%
YP	*R. cliffordpopei* (0.168523), *H. tchangi* (0.128824), *H. sauvagei* (0.106099)□, *S. obscura* (0.085890), *M. kiatingensis* (0.059258)□, *C. carpio* (0.058180), *P. crassilabris* (0.058116), *S. sichangensis* (0.048004)□, *S. argentatus* (0.042231), *C. mongolicus* (0.035671), *S. undulata* (0.035238), *S. meridionalis* (0.031907), *S. davidi* (0.031284)□, *Acipenser* sp.× *Acipenser* sp. (0.023719)	14	28.57%
FL	*H. tchangi* (0.274556), *C. mongolicus* (0.197472), *C. carpio* (0.058304), *S. davidi* (0.050213)□, *R. cliffordpo*pei (0.039998), *S. sichangensis* (0.026105)□, *S. curriculus* (0.025957), *S. superciliaris* (0.022193)□, *S. obscura* (0.021626), *P. parva* (0.021457)	10	33.33%
Mainstream overall	*H. tchangi* (0.417195), *C. carpio* (0.178973), *R. cliffordpopei* (0.097563), *O. bidens* (0.085186)□, *I. punctatus* (0.023639), *C. zillii* (0.022276)	6	16.67%

*Note*: □: Rheophilic fish species.
